# Surgical resection of cerebellar hemangioblastoma with enhanced wall thickness: A report of two cases

**DOI:** 10.3892/ol.2015.2951

**Published:** 2015-02-10

**Authors:** ZHENXING SUN, DAN YUAN, YAXING SUN, PENGXIANG YAN, HUANCONG ZUO

**Affiliations:** 1 Department of Spinal Cord and Spine Neurosurgery, Yuquan Hospital, Medical Center, Tsinghua University, Beijing 100049, P.R. China; 2 Department of Nephrology, The Luhe Teaching Hospital of the Capital Medical University, Beijing 101149, P.R. China; 3 Department of Psychiatry, The Second Municipal Hospital of Zaozhuang City, Zaozhuang, Shandong 277218, P.R. China; 4 Department of Neurosurgery, Tiantan Hospital, Capital Medical University, Beijing 100050, P.R. China

**Keywords:** hemangioblastoma, cerebellar, surgical experiences

## Abstract

Hemangioblastomas are tumors of the central nervous system, and the cerebellum is the most common site of occurrence. Cerebellar hemangioblastoma with enhanced wall thickness is rare and often misdiagnosed preoperatively. At present, no unified radiological classification system based on magnetic resonance imaging (MRI) findings exists for cerebellar hemangioblastoma, and this tumor type can be solid or cystic mass, according to the MRI findings. The most common presentation of cerebellar hemangioblastoma observed radiologically is a large sac with small nodules, where the wall of the large cyst is not enhanced. A tumor with enhanced large cysts and tumor nodules is extremely rare. The most effective treatment is complete resection of the cyst and the solid growth. The present case reports the successful treatment of two cases of cerebellar hemangioblastoma with enhanced wall thickness, including the MRI findings for the differential diagnoses and the surgical experiences.

## Introduction

Hemangioblastomas are tumors of the central nervous system that most frequently arise from the vascular system; they are classed as is WHO grade I tumors (1). In adults, 7–10% of tumors arise in the posterior fossa (2) and the cerebellum is the most common site of occurrence (3,4). As a number of features may be observed on magnetic resonance imaging (MRI), (1,3–6) according to previous reports, cerebellar hemangioblastoma can be predominantly divided into two categories on the basis of MRI findings. The most common radiological presentation of cerebellar hemangioblastoma is a large sac or cyst and small tumor nodules. We hypothesize that this type may be further divided into two subtypes: One which exhibits no enhancement at the wall of the large cyst, but with evenly enhanced tumor nodules; and another with an enhanced large cyst and tumor nodules. The less common type of cerebellar hemangioblastoma is a solid mass, and also comprises two subtypes: One type contains multiple solid tumors and exhibits homogeneous enhancement on MRI; the other subtype is a solid tumor with single or multiple cysts, where the solid portion is enhanced and the cystic region is non-enhanced (7). In addition to the two main tumor types, the rarest variant of this tumor exhibits an enhanced cyst wall, based on the cystic nodules, and is accompanied by enhanced uneven walls (6). The first type (a large sac or cyst with small tumor nodules) has surrounding edema. The other two types (one type is a solid mass, one type exhibits enhanced cyst wall) exhibit an obvious mass and do not have surrounding edema. However, in spite of these characteristic features on imaging, in the preoperative and differential diagnoses, solid cerebellar hemangioblastoma and nodular cerebellar hemangioblastoma with enhanced wall are often misdiagnosed as high-grade gliomas (6).

For cerebellar hemangioblastoma with an enhanced cystic wall, surgical resection is the most effective treatment. The tumor is unlikely to recur following complete resection, therefore chemotherapy or radiotherapy is not usually required. Cerebellar hemangioblastoma exhibits a good prognosis following complete resection, with a five-year survival rate of >50% (2). The current study presents two cases of cerebellar hemangioblastoma, which both exhibited enhanced wall thickness. Written informed consent was obtained from both patients.

## Case reports

### Case one

**Figure 1:**
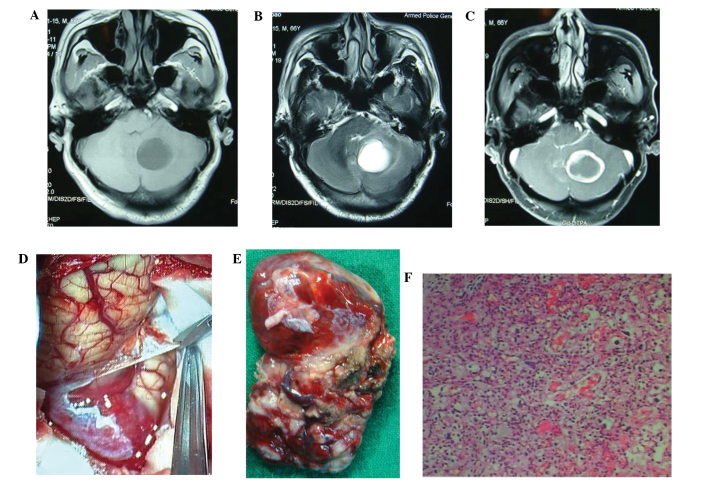
Patient 1: A cystic mass was in the right cerebellar hemisphere. (A) The mass was hypointense on T1-weighted images. (B) The mass was hyperintense on T2-weighted images. (C) The mass showed an enhanced solid portion of the wall of the mass after injection of gadolinium-diethylenetriaminepentaacetate. (D) The mass was located in the brain parenchyma with white calcification under microscope. (E) The mass was completely resected. (F) Hematoxylin and eosin staining confirmed the diagnosis of cerebellar hemangioblastoma (magnification, ×100).

A 66-year-old male, presented to the Department of Neurosurgery, Tiantan Hospital, Capital Medical University (Beijing, China) with the primary complaint of an intermittent headache for four months, with two months of ataxia. Physical examination showed abnormal ataxia in the right extremities. MRI revealed a cystic mass in the right cerebellar hemisphere. The mass was hypointense on T1-weighted images, hyperintense on T2-weighted images, and showed an enhanced solid portion on the wall of the mass following the injection of gadolinium-diethylenetriaminepentaacetate (Fig. 1A, B, C). The patient was treated using the suboccipital approach under general anesthesia; two thick draining veins were identified at the surface of the tumor with multiple, thick feeding arteries surrounding the tumor, all of which were closely adhered to the surrounding tissues. The tumor boundary was clear, and the upper region, located in the brain parenchyma, was cystic (Fig. 1D). The cyst fluid was light yellow and transparent, with a volume of ~5 ml. After cutting the wall membrane, the cluster of red vasular masses with abundant blood supply was evident. The tumor boundary was separated by occluding the blood supply and blocking the draining veins. The complete resected mass was ~2×3×3 cm size (Fig. 1E). Hematoxylin and eosin (HE) staining confirmed the diagnosis of hemangioblastoma (Fig. 1F). Postoperatively, the headache completely regressed and the ataxia subsided. Follow-up was conducted annually and three years after surgery the patient was asymptomatic. At the time of writing the patient was well, with no evidence of recurrence.

### Case two

**Figure 2:**
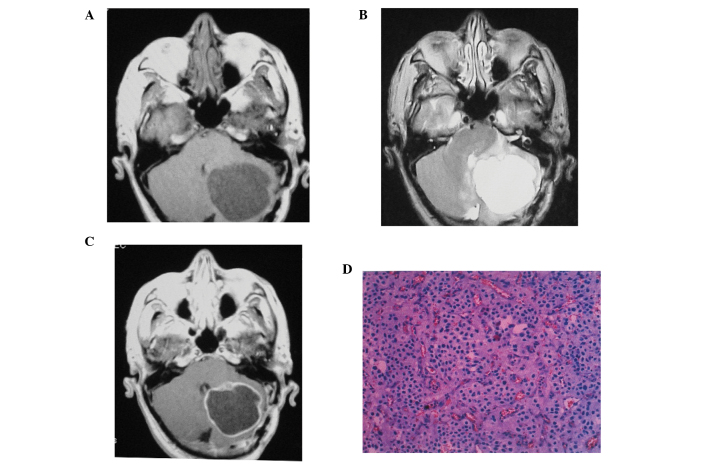
Patient 2: A cystic mass was in the left cerebellar hemisphere and vermis. (A) The mass was hypointense on T1-weighted images. (B) The mass was hyperintense on T2-weighted images. (C) The mass was enhanced irregular nodular lesion without obvious edema after injection of gadolinium-diethylenetriaminepentaacetate. (D) Hematoxylin and eosin confirmed the diagnosis of cerebellar hemangioblastoma (magnification, ×100).

A 60-year-old male was admitted to the Department of Neurosurgery, Tiantan Hospital, Capital Medical University, with gait ataxia, headaches, nausea and vomiting for one month. Physical examination showed abnormal ataxia in the left extremities with gait ataxia. Subsequently, MRI revealed irregular long T1/T2 signals in the left cerebellar hemisphere and vermis, and enhanced irregular nodular lesions without obvious edema (Fig. 2A, B, C). Surgery was performed to remove the lesions, during which, a red, medium-texture, nodular tumor with a rich blood supply was identified. The cyst fluid was pale yellow with a volume of ~8 ml. For the complete resection of the tumor, it was fully separated from the surrounding tissue. On removal, the tumor measured 1.5×1×1 cm in size. As with the previous case, HE staining confirmed the diagnosis of hemangioblastoma (Fig. 2D). Two years after surgery the patient was asymptomatic, with no evidence of recurrence.

## Discussion

Cerebellar hemangioblastoma is the most common form of hemangioblastoma (3,4). Based on MRI findings, there are several known types of this tumor. The most common type consists of small nodular tumors on the side of a large cyst and the two rarer types comprise a solid mass, or a lesion with an enhanced cyst wall due to cystic nodules, which exhibits enhanced uneven walls on imaging (2).

Surgical resection is the most effective treatment for cerebellar hemangioblastomas with an enhanced cystic wall (8). However, for this type of lesion, the tumor must not be punctured, biopsied or blocked via resection due to the rich blood supply. The enhanced tumor wall indicates that it contains partial tumor cells, therefore to avoid recurrence of the tumor, the wall and the solid part of the tumor require total resection (9). This type of tumor has a benign characteristic, and is generally located in the brain parenchyma (9). However, tumors may perforate the surface of the brain and metastasize to the surrounding regions (10). Following complete resection, it is unlikely that the tumor will recur, and therefore chemotherapy or radiotherapy is not frequently required (9). Even in cases where residual tumors are identified, only a small number of these tumors become malignant.

According to our experience regarding surgical procedures, in order to achieve separation of the lesion from the surrounding tissue in the present cases, initially the feeding artery was occluded until the surface tension decreased, and subsequently the draining veins were blocked (11). The tumors were then resected completely. Multiple feeding arteries are often present, as well as more than one abnormally thick draining vein, with large diameters and thick walls (9), which were identified in the two patients of the present cases. Occasionally, a localized flow and rich blood supply within the tumor is observed and the color of intravenous blood is bright red (9), which occurred in the present cases. However, caution must be taken not to block the draining veins mistakenly based on the color of the blood, as this may result in heavy bleeding due to the venous return obstruction.

In the present cases the tumors were resected successfully. No subsequent treatment was required following surgery, and a full recovery was achieved. After three years of follow-up, the patients were asymptomatic and at the time of writing, no recurrences had been identified. Continued follow-up of the two patients has been planned. The limitations of the current study included the small number of cases presented and the short follow-up periods.
